# *Melaleuca armillaris* Essential Oil as an Antibacterial Agent: The Use of Mesoporous Bioactive Glass Nanoparticles as Drug Carrier

**DOI:** 10.3390/nano13010034

**Published:** 2022-12-21

**Authors:** Josefina Ballarre, Daniel Buldain, Irem Unalan, Juan I. Pastore, Nora Mestorino, Aldo R. Boccaccini

**Affiliations:** 1 Material’s Science and Technology Research Institute (INTEMA), UNMdP-CONICET, Av. Colón 10850, Mar del Plata B7600, Argentina; 2 Pharmacologic and Toxicological Studies Laboratory (LEFyT), Veterinary Science Faculty, UNLP, La Plata B1900, Argentina; 3 National Council of Scientific and Technical Research (CONICET), Argentina; 4 Department of Materials Science and Engineering, Institute of Biomaterials, University of Erlangen-Nuremberg, Cauerstrasse 6, 91058 Erlangen, Germany; 5 Digital Image Processing Laboratory ICyTE, Universidad Nacional de Mar del Plata (UNMdP), Argentina

**Keywords:** mesoporous bioactive glass, essential oil, antibacterial properties, *Melaleuca armillaris*

## Abstract

Bioactive glasses have been proposed for bone tissue engineering due to their excellent biocompatibility and osteo-inductive behaviour. The generation of mesoporous bioactive glass (nano) particles adds a high surface area for the dissolution and release of bioactive ions, and the possibility to load them with different drugs for antibacterial purposes. Essential oils (EO) are an interesting resource for alternative medical therapy, providing antimicrobial compounds that come from organic/natural resources like aromatic plants. Also, a biological polymer, such as chitosan, could be used to control the release of active agents from mesoporous bioactive glass (MBG) loaded particles. This work presents MBG particles with nominal composition (in mol) 60% SiO_2_, 30% CaO and 10% P_2_O_5_, loaded with essential oil of *Melaleuca armillaris*, which contains 1,8-cineol as the main active component, with an inhibitory in vitro activity against several bacterial species. Also, co-loading with a broad-spectrum antibiotic, namely gentamicin, was investigated. The MBG particles were found to be of around 300nm in diameter and to exhibit highly porous open structure. The release of EO from the particles reached 72% of the initial content after the first 24 h, and 80% at 48 h of immersion in phosphate buffered solution. Also, the MBG particles with EO and EO-gentamicin loading presented in vitro apatite formation after 7 days of immersion in simulated body fluid. The antibacterial tests indicated that the main effect, after 24 h of contact with the bacteria, was reached either for the MBG EO or MBG EO-gentamicin particles against *E. coli*, while the effect against *S. aureus* was less marked. The results indicate that MBG particles are highly bioactive with the tested composition and loaded with EO of *Melaleuca armillaris*. The EO, also combined with gentamicin, acts as an antibacterial agent but with different efficacy depending on the bacteria type.

## 1. Introduction

Ceramics used for bone repair and reconstruction are called bioceramics. They can be bioinert, resorbable, bioactive, and they can be combined with polymers to develop scaffolds for tissue engineering applications [1]. In the bioactive bioceramics family, bioactive glasses (BGs) are widely used because of their excellent properties, in particular interaction with the biological environment based on the possibility of releasing functional ions, which are biologically active [2,3,4]. Hench et al. developed the 45S5 BG (45 SiO_2_-24.5 Na_2_O-24.5 CaO-6 P_2_O_5_ (wt%)), in the 1970s, the first material showing strong bond to living tissues [5]. This glass is capable to react and interact with the human physiological medium, leading to new tissue formation [6]. The dissolution products of BGs induce rapid mineralization of the tissue because of the calcium (Ca) and phosphate ions released acting as bone cells catalyzers [4,7]. Also, the presence of Si species has been proved to regulate gene expression of osteoblastic cells [8] and also to enhance angiogenesis [4,9].

A wide range of bioactive glass compositions, suitable for bone tissue engineering and regeneration, can be produced by the melt quenching method (traditional process), and sol-gel techniques [3]. The advantage of sol-gel processing resides in the homogeneity of the system, the wide range of compositions possible and the different shapes or microstructures that could be obtained [10]. One convenient type of BG is in the form of nanoparticles. Sol-gel derived bioactive glasses have improved biological reactivity (in comparison to melt-derived BGs) since they have a higher surface area and the presence of silanol groups, which can act as nucleation sites for the formation of calcium phosphate bioactive compounds [11]. In addition, a sol-gel modified process, with a polymeric template, can be used to synthesize mesoporous bioactive glasses (MBGs), which combine the properties of an open sol-gel glass structure matrix with high specific surface area and large pore volume and exhibit an ordered pore structure. These last features can lead to the loading of MBG particles with different therapeutic agents, such as gentamicin [10], to be a potential controlled drug delivery system [12]. In addition, the control of this release could be designed by adding an organic or degradable coating to the particles, such as chitosan. The use of this biopolymer in the biomedical field has become more significant exploring (the well-known properties of this chitin-derived polysaccharide) [13].

Antimicrobial resistance is a relevant issue in hospital related pathologies requiring enhanced efforts for the prevention and treatment of implant associated infections [14]. Essential oils (EOs) are an interesting resource for alternative therapeutic approaches as they contain antimicrobial compounds derived from organic/natural resources like aromatic plants. The active agents or components of EOs can act as bacteriostatic or bactericides depending on the different situations, responding to several action mechanisms with a wide variety of target sites [15]. This process leads to the destabilization of the phospholipid bilayer, representing the disruption of the bacteria membrane, the loss of bacterial intracellular components, and inactivation of enzymatic mechanisms [16]. Genus *Melaleuca* belongs to the Myrtaceae family, which includes several species of plants producing EOs. *Melaleuca armillaris*, is one of the most widely cultivated *Melaleuca* plants [17]. *M. armillaris* EO gas chromatography coupled with mass spectrometry spectrum revealed the presence of 1,8-cineol as the main component, which has inhibitory in vitro activity against several bacterial species, including *S. aureus* [16,18].

The aim of this work is to synthesize, characterize and test mesoporous silica-based bioactive glass particles, as carriers for *Melaleuca Armillaris* EO and gentamicin, for the prevention of intrahospital infections. Combination of BGs and phytotherapeutic agents has been explored in the past, as particles or composites [19,20], including the utilization of MBG nanoparticles to carry and delivery plant-derived compounds [21], but there has been limited work on EO/MBG systems. The MBG particles in this study were tested against *E. coli* and *S. aureus* and their bioactivity as apatite precursors for bone repair was also determined. To the best of our knowledge, this is the first study investigating mesoporous silica-based bioactive glass sub-micrometric particles combined with *Melaleuca Armillaris* EO and gentamicin to impart antibacterial activity and to create a potential synergetic effect between the EO, gentamicin and the release of MBG ions for antibacterial bone regeneration. 

## 2. Materials and Methods

### 2.1. Melaleuca armillaris Essential Oil Extraction

The collection of leaves and herbaceous branches was carried out in Coronel Brandsen, Buenos Aires, Argentina (latitude 35°06′18.9″ S and longitude 58°10′57.0″ W). EO was obtained as was previously reported by steam distillation of the whole collected fresh biomass and its composition was analyzed by gas chromatography with mass detection and flame ionization, revealing the presence of 1.8 cineol (72.3%) and limonene (7.8%) and α-pinene (6.0%) as the main components [16]. Subsequently, the EO was dried with sodium sulphate anhydrous at room temperature, filtered with a cotton funnel, and stored at 4° C in an amber glass bottle.

### 2.2. Synthesis of Mesoporous Bioactive Glass (MBG) Particles

Tetraethyl orthosilicate [TEOS]—99% (Sigma), triethyl phosphate [TEP]—99% (Aldrich, Germany), and calcium nitrate [Ca(NO_3_)_2_.4H_2_O]—98% (Aldrich, Germany), were used as silicon, phosphorous and calcium sources, respectively. Furthermore, ethyl acetate, cetyltrimethylammonium bromide [CTAB]—(Merck, Germany), ammonium hydroxide 28% (VWR, France) and distilled water (MilliQ) were used. 

MBG particles (nominal composition 60% SiO_2_, 30% CaO, 10% P_2_O_5_; in mol percentage, were produced using a modified Stoeber process [6,22]. Firstly, 1.4 g CTAB (soft template) was dissolved in 66 mL of deionized water under continuous stirring. Then, 20 mL ethyl acetate was poured dropwise into the previous solution and the mixture was stirred for 30 min. After that time, ammonium hydroxide solution (28%) was added to maintain pH at 10.5 and 7.2 mL of TEOS was incorporated into the mixture to generate the mesoporous particles under continuous stirring. Finally, the sources of calcium (3.84 g) and phosphorous (1.84 mL) were added step-wise, followed by magnetic stirring for 4 h to let the components to react. The generated suspension was centrifuged at 7830× *g* rpm (Centrifuge 5430R, Eppendorf, Germany) for 10 min to separate particles. Two water and one ethanol washes were also done. Afterward, the obtained particles were dried in an oven at 60 °C overnight, and then calcinated at 700 °C (heating rate of 2 °C/min) for 3 h.

### 2.3. EO and Drug Load into MBG Particles

*Melaleuca armillaris* essential oil (EO) and gentamicin sulphate (Ge) (Merck, Germany) were used as antibacterial agents. The MBG particles were loaded with either EO or Ge, and with a mixture of both (EOGe). Ethanol solutions with 40 µL/mL of EO, other with 40 µg/mL of Ge and another combining 20 µL/mL of EO, and 20 µg/mL of Ge were prepared. 0.5 g of MBG particles were immersed in 50 mL of each one of the prepared solutions for 6 h under continuous stirring. After that, the suspensions were centrifuged at 4800× *g* rpm for 10 min and dried at 60 °C overnight.

### 2.4. Coating of Loaded MBG Particles

A chitosan layer was deposited onto the MBG-loaded particles by applying a previous protocol [23], in order to produce a controlled drug delivery behaviour. Chitosan (high molecular weight, 90–95% degree of deacetylation, Ghion SA, Argentina) was assembled onto the particles’ surface in a concentration of 1 mg/mL in water. The pH value of the electrolyte solution was adjusted to 5 by the addition of either acetic acid or NaOH. The deposition time was 15 min, and then the particles were washed with DI water, centrifuged at 4800× *g* rpm for 3 min, and again dried at 60 °C overnight.

### 2.5. Particles Characterization

The morphology of the synthesized MBG loaded and unloaded particles was analyzed by scanning electron microscopy (SEM, Auriga ZEISS SNr. 4570, Carl Zeiss Microscopy) at an energy of 5 kV. ATR-Fourier Transform Infrared spectroscopy (Shimadzu IRAffinity-1S, Shimadzu Corp., Japan) was carried out in transmission mode at the wavenumber ranging from 4000 to 400 cm^−1^ at a resolution of 4 cm^−1^. Verification of the glassy state of MBG particles was carried out by X-ray diffraction (XRD) analysis in a PANalytical diffractometer with Cu-Kα radiation at 40 kV and 30 mA, in a 2θ range between 20° and 80° at a scan rate of 1°/min.

Digital Image Processing (DIP) techniques were used to analyze and quantify the particles’ shape and distribution prior to deposition. Image analysis techniques have been widely used not only for size but also for particle shape characterization [24,25,26]. As in [26], in this work, MBGs size distribution was analyzed using an adaptation of the algorithm proposed by Meng et al. [27]. This algorithm is mainly based on local adaptive Canny-edge detection and the Hough Transform used to find ellipses. An ellipse can be described by its center coordinate, major axis length, minor axis length, and orientation. This algorithm finds the minor and major axis lengths of every object (inside the MBGs particles) present in the image. The MBG particles size distribution is defined as the discrete function p(dk) = nk, (dk is the kth diameter measured and nk is the number of particles with that diameter, either major or minor) [24]. The number of particles detected for each of the conditions was sufficient to determine the particle size distribution [28]. 

### 2.6. EO Encapsulation Percentage/Release

The concentration of *M. armillaris* EO in ethanol solution was monitored by UV-VIS spectrophotometer (Specord 40, Analytic Jena, Germany) at 320 nm. After particle loading, with the 6 h reaction, EO and EOGe-loaded mesoporous bioactive glass particles were centrifuged at 4800× *g* rpm and the supernatant was removed. The amount of EO loaded into MBG particles was measured by the change in the UV intensity before and after loading. The EO loading efficiency (*E*) was calculated by using the equation:

(1) $$Loading\;efficiency\;\left(E\right)\left(\%\right)=\frac{Initial\;concentration-Supernantant\;concentration}{Initial\;concentration}\times 100$$

The release of *M. armillaris* EO from MBG particles, with and without the addition of gentamicin, was studied in phosphate buffer solution (PBS) (pH 7.4). For the release experiments, 60 mg of MBG EO or MBG EOGe particles, as well as the same particles coated with chitosan, were immersed in 2.5 mL of PBS at 37 °C for 14 days. At each time point (such as 1 h, 2 h, 3 h, 5 h, 8 h, 24 h, 2 d, and up to 14 d), samples were centrifuged at 4000× *g* rpm for 3 min and the supernatant was removed and refreshed with 2.5 mL of fresh PBS. Sample solutions were stored at −20 °C for testing later. The EO release was determined by a UV spectrophotometer at 250 nm and the amount of EO was calculated against a calibration curve with R = 0.99. The cumulative release of EO (in μL/mL) was obtained by adding the amount of EO obtained at each time point, calculated by the previous calibration curve. The experiments were carried out in quadruplicate.

### 2.7. In Vitro Apatite Formation Ability

Acellular bioactivity studies were performed using cylindrical samples (10 mm diameter, 1 mm high) obtained by pressing 30 mg of MBG at an uniaxial pressure of 4 MPa (hydraulic press, PE-010, Mauthe Maschinenbau) for 1 min. The samples surface reactivity was investigated by detecting the possible formation of an apatite-like layer on the surface of the MBG disks after immersion in simulated body fluid (SBF) [29]. MBG disks were immersed for 24 h, 7 and 14 d in SBF at 37 °C, pH 7.4, according to [30]. Briefly, the volume used (Vs, mL) was calculated to keep the ratio VS = DS/0.075 constant, where DS is the external geometric area (cm^2^) of the sample. After the immersion time, disks were removed and rinsed with ethanol, and dried at 60 °C in air. The inorganic bioactivity, i.e., apatite-like compounds formation ability, was analyzed by FTIR and SEM.

### 2.8. Antibacterial Behaviour

The antibacterial effect of the MBG particles with EO and with the combination of EO and Gentamicin, against *S. aureus* (Gram-positive) and *E. coli* (Gram-negative) bacteria, was evaluated by relative bacterial viability assay [31] and Agar Diffusion method [32]. MBG particles without an antibacterial agent were also used for comparison. The MBG-loaded particles were sterilized under UV light for 60 min on a sterile bench. Bacteria were grown in lysogeny broth (LB, Luria/Miller) medium at 37 °C for 24 h. Then, the bacterial optical density (*OD*) was arranged to 0.015 (approximately 1 × 10^7^ colony forming units per mL) at 600 nm, using a spectrophotometer (Thermo Scientific GENESYS 30, Germany). MBG powders were incubated in LB medium (100 mg/10 mL) for 24 h at 37 °C under continuous shaking. After the glass separation from the LB medium by filtration, the bacterial suspension with a volume of 20 μL was added to 2 mL of the obtained elution extract. All samples were incubated at 37 °C for 6, 24 and 48 h. The relative viability of the bacteria was calculated according to the following equation:

(2) $$\mathrm{Relative}\;\mathrm{bacteria}\;\mathrm{viability}\;\left(\%\right)=\frac{OD\;sample}{OD\;control}\times 100$$

The agar diffusion method was performed with the bacterial suspension prepared as described above. The agar plates were prepared as described in previous work [30]. Briefly, LB agar was poured into Petri dishes, and 20 μL of 0.015 *OD* bacterial suspension was spread on the top of the agar plate. Pellets of MBG particles (7 mm diameter and 30 mg) with and without antibacterial agents (hydraulic press, PE-010, Mauthe Maschinenbau, 1 MPa for 1 min) were placed on top and centre of the agar plates and incubated for 24 and 48 h at 37 °C, against control groups without pellets. The experiments were carried out in triplicate. The MBG pellets’ antibacterial activity was determined by measuring optical changes in the density of the bacterial colonies in the surrounding regions. The inhibition zones’ presence and size, defined as areas free of bacteria, were measured optically.

## 3. Results and Discussion

**Figure 1:**
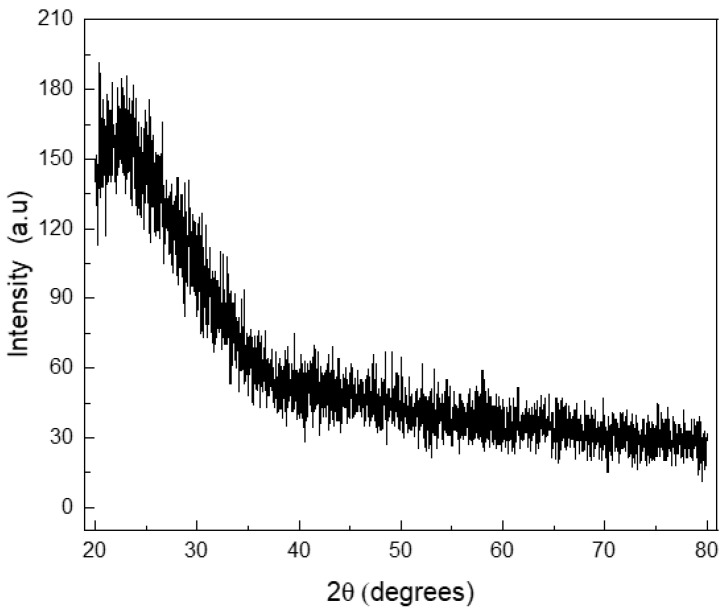
X-ray diffraction pattern for the mesoporous bioactive glass particles synthetized.

Silicate MBG particles were obtained by a simple and moderate cost technique, using standard equipment and non-toxic reagents/solvents. Cetyltrimethylammonium bromide (CTAB), used as a cationic surfactant for the mesoporous matrix formation, is a toxic agent [33], but it was washed after MBG particle formation and the generated particles were proven to be non-cytotoxic [6,22]. The MBG particles were expected to be glassy (non-crystalline): this state was corroborated by XRD essay (Figure 1). 

**Figure 2:**
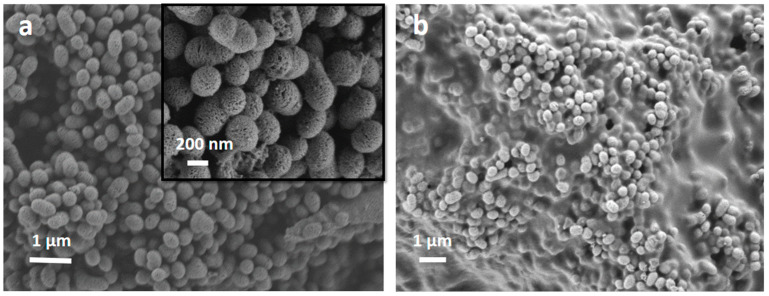
Scanning electron microscopy images of the MBG EOGe particles denoting their porous structure (**a**) and the MBG EOGe particles with chitosan coating showing homogeneous particle size (**b**).

The size and morphology of the SiO_2_-CaO-P_2_O_5_ MBG particles were analyzed by SEM and DIP processing. They showed a homogeneous mesoporous structure with a large surface area/volume ratio [6,34]. Both MBG and MBG EO (and MBG EOGe) particles presented the same shape and structure, denoting no alterations with the EO and gentamicin incorporation, as can be seen in Figure 2a. When the chitosan coating treatment was applied, it was not obtained for each particle individually, but as a coating or “glue”, covering some of them simultaneously (Figure 2b). 

**Figure 3:**
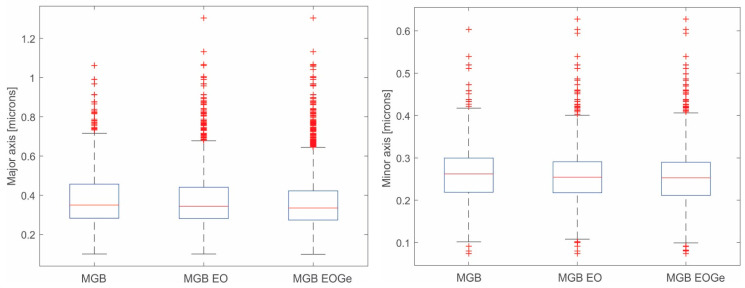
Box-plots of the size distribution of the MBG, MBG EO and MBG EOGe particles. (**Right**), minor diameter; (**left**), major diameter.

**Figure 4:**
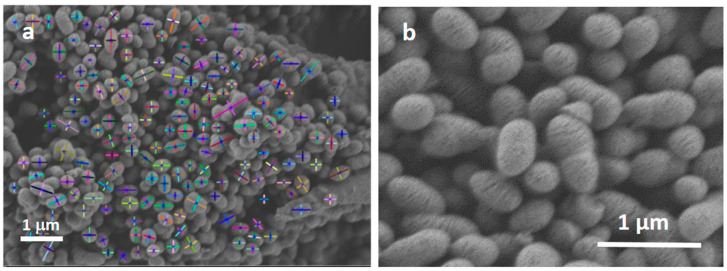
(**a**) Major and minor diameters of the MBG EOGe particles measured by DIP, (**b**) coalesced MBG particles.

The size and distribution of the MBG particles are shown in the box-plots in Figure 3. The plots reveal particle distributions with a slight asymmetry and some scattering for the different conditions. This data scattering could be associated with errors in the segmentation process or due to the synthesis process of the MBG particles: if the nuclei are not spread enough, the MBG particles will growth together, generating an ovoid-shape particle. This is the reason why minor and major diameters were measured by DIP for the obtained particles. Figure 4a shows an image example of the measurements by DIP, with the major and minor diameters of the MBG EOGe particles. Also, Figure 4b shows the coalescence of several particles to form one ovoid-like shape particle. After DIP analysis was carried out, a manual “cut” post treatment was done to take out of the measurement most of the coalesced particles. The threshold value was set at 0.6 μm. For this set of filtered values, the media values and the associated errors of MBG, MBG EO and MBG EOGe are calculated and listed in Table 1. From the obtained statistics, it can be concluded that the load of the MBG particles either with EO or EOGe after particle synthesis does not affect the final shape and size of the particles.

**Figure 5:**
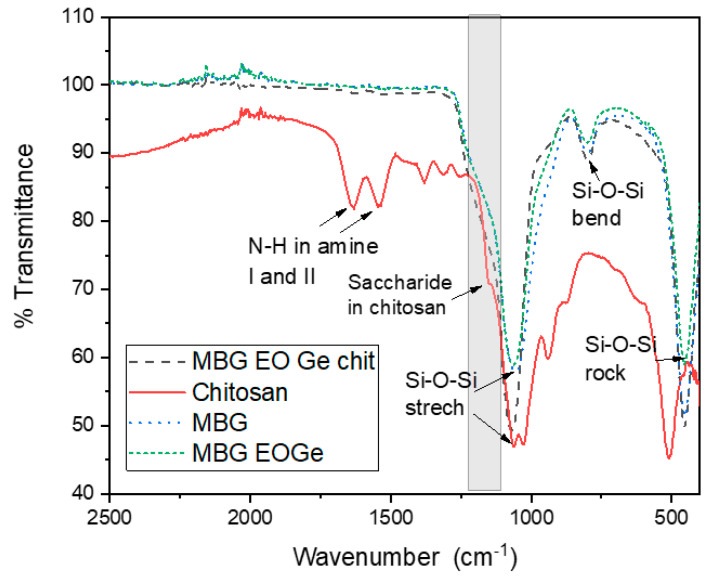
ATR-FTIR characterization of the mesoporous bioactive glass particles (MBG) with *Melaleuca armillaris* EO (MBG EO, with EO and gentamicin (MBG EOGe), and with EO and gentamicin cover with chitosan (MBG EOGe chit). Also, chitosan was analysed.

The chemical characterization (internal bonding) of the MBG particles loaded with and without chitosan particles was carried out by ATR-Fourier Transformed Infrared Spectroscopy, in transmittance mode (Figure 5). The typical bands of 1056 cm^−1^ (Si-O-Si stretching), 795 cm^−1^ (Si-O-Si bending) and 453 cm^−1^ (Si-O-Si rocking) of silica-based bioactive mesoporous particles were found for MBG and MBG EOGe particles [6,22]. The presence of the high degree of deacetylation chitosan made a shift in the 1056 cm^−1^ band to 1068 cm^−1^. Also; there are two bands with a slight shift in the MBG FTIR spectra due to the chitosan presence: 1220–1106 cm^−1^ and 1015–870 cm^−1^, both related to the bands of the saccharide structure of chitosan (1152 and 938 cm^−1^) [35,36].

The loading efficiency of the EO in the MBG particles with or without the presence of Ge was calculated using a UV-Vis method. The value found for EO was 96.1 ± 0.4% in the MBG EO particles, and 86.7 ± 1.6% of EO loading efficiency for the MBG EOGe ones. High values of incorporation of EOs can be obtained with similar methods on mesoporous particles [37,38]. For the particles also containing Ge, the method is not so accurate because the gentamicin UV-Vis band (350 nm) may interfere with one of the essential oil bands in ethanol (320 nm).

**Figure 6:**
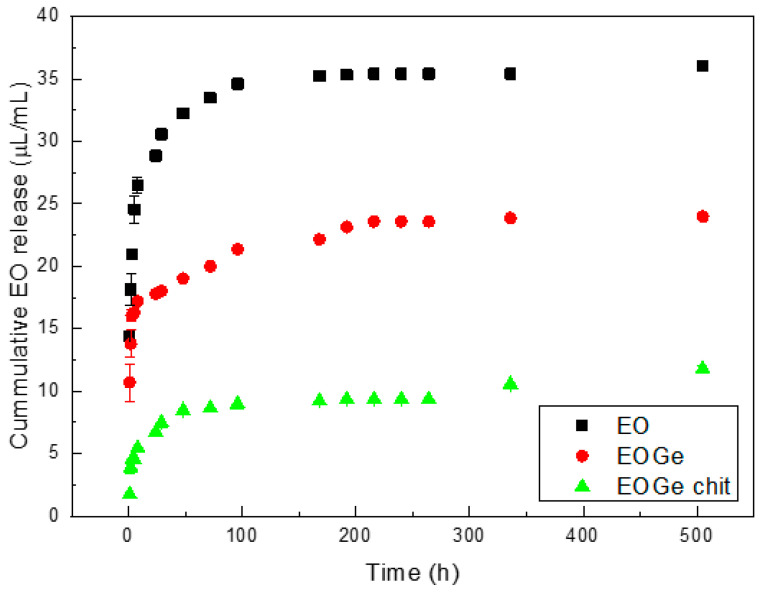
Release of EO (μL/mL) from MBG EO, MBG EOGe and MBG EOGe chit particles in PBS.

Figure 6 shows the release of EO as function of time from the MBG EO, MBG EOGe and MBG EOGe covered with chitosan particles in PBS. The release value is in micrograms per milliliter of PBS. The initial loading concentration of the MBG EO particles was 40 μL/mL, and for the MBG EOGe, the concentration was 20 μL/mL of the EO and the same for gentamicin. The absorbance of the EO in the UV-Vis spectrum in the case of the MBG EOGe particles is affected by the absorbance of gentamicin, which in PBS has a diffuse band between 200 and 250 nm, without any complexing agent [27]. This effect is enlarging the total amount of cumulative release from the MBG EOGe particles, relating to the EO. 

The tendency of the release kinetics for the three kinds of particles is with a fast release in the first 20 h of immersion in PBS, and then for the next 30 h a moderate release, leading to a plateau. This is a common release behaviour for drug loaded mesoporous silica based particles [37,39]. It is worthy to point out that in all loaded systems, the release curves are influenced by interactions between the drug and the particle. As discussed in the literature, the drug–pore wall attractions may represent the primary reason for the commonly observed dependence of drug release kinetics from such porous structure [40]. The slight EO-MBG binding can be denoted by the release performance in this work: after 48 h of immersion, the cumulative release is up to 80%, and the final cumulative release for the MBG EO particles is 90.1 ± 14.5% after 21 days of immersion in PBS. In the case of MBG EOGe and MBG EOGe chit, the cumulative release value is higher than expected (in the case of MBG EOGe, higher than the initial particle loading of 20 μL/mL), due to the effect of the gentamicin presence in the UV-Vis absorbance band. Nevertheless, it can be stated that the effect of the chitosan coating reduces the release of the essential oil by more than 50%, being the tendency after 24 h 17 μL/mL vs. 7 μL/mL. After 21 days of immersion, no significant release is shown for the MBG EOGe chit system, comparing with the one without chitosan coating. The objective of adding a chitosan coating to the MBG particles was to retard in a short period of time the release of antibacterial agents, but not to block the release in long periods of time.

The release kinetics of the essential oil from the mesoporous particles was studied from the results shown in Figure 6. The kinetic behavior of the EO release from MBG particles was described considering the reported assumption of a pseudo second-order model. This kinetic model is based on the assumption that the rate-limiting step is chemical sorption or chemisorption and predicts the behavior over the whole range of adsorption/release [41]. The equation is:

(3) $$\frac{dQ_{t}}{dt}=k_{2\;}{\left(Q_{e}-Q_{t}\right)}^{2}$$

or in other words:

(4) $$q_{t}=\frac{q_{e\;}^{2}k_{2}t}{1+q_{e}k_{2}t}$$

where Q_t_ (μL/mL) is the adsorbed ions at time *t* (hours), Q_e_ is the quantity of adsorbed ions after equilibrium t (μL/mL) and k_2_ is the model rate constant (mL/μL. hour). It is possible to linearly adjust the mechanism and calculate the constants. From the EO and EOGe release, with and without chitosan presence, the values are shown in Table 2. A very good correlation with the proposed mechanism for the release of agents from mesoporous materials can be observed [42].

The in vitro performance of the synthesized mesoporous bioactive glass particles is important to analyze surface properties such as bioactivity and degradation in simulated body fluid. It is well known that with a higher surface reactive area, the ion exchange between the MBG particles and the medium is higher. Moreover, more reaction is possible as pore size and structure, are also controlled. This issue could be strongly dependent on the composition and sol-gel synthesis parameters. Stable mesoporous particles with SiO_2_ content from 60 to 90% can be formed, independently of CaO or P_2_O_5_ content [43].

**Figure 7:**
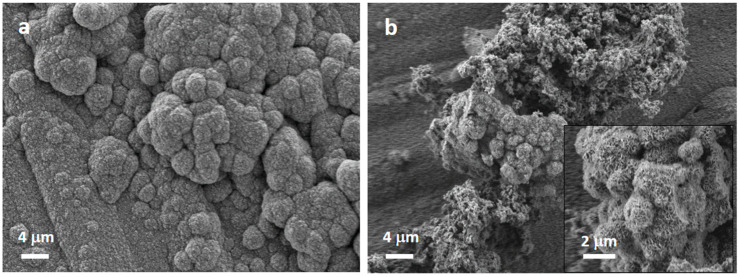
Scanning electron microscopy images of pellets of MBG (**a**) and MBG EOGe (**b**) after 7 days immersion in SBF.

**Figure 8:**
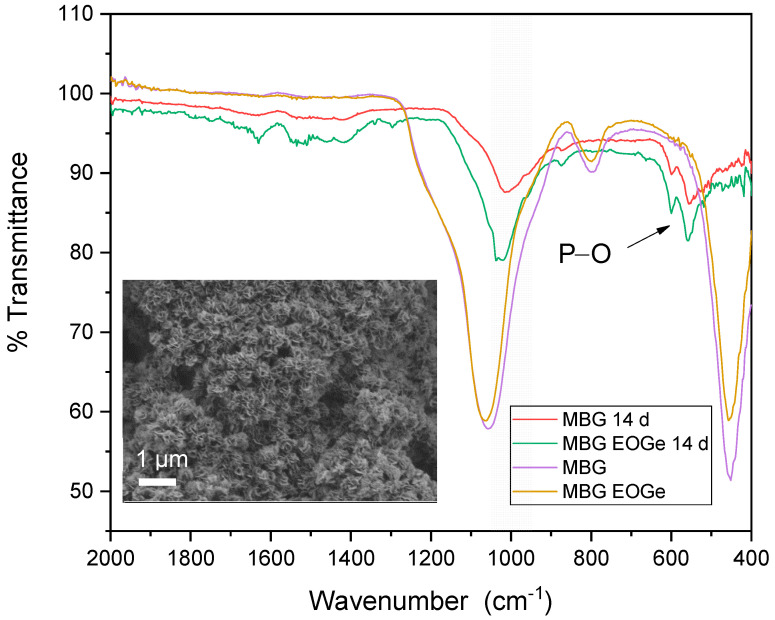
ATR-FTIR spectra of the MBG and MBG EOGe pellets before and after 14 days of immersion in SBF.

Regarding inorganic bioactivity or bioreactivity, the synthesized MBG particles without and with EO and EOGe loading, were tested as pellets immersed in SBF, to analyze hydroxyapatite formation, which is the indicator of bioactivity of BGs [44]. Figure 7 shows SEM images indicating the formation of globular-acicular-like deposits on the surface, the classical shape of apatite formations in vitro [45]. The effect was found in MBG, MBG EO and MBG EOGe pellets, after 7 days of immersion in SBF. The MBG samples showed more spread and larger deposits, but after 14 days, all surfaces were covered equally with globular and acicular deposits. FTIR spectra are shown in Figure 8, to analyze the presence of calcium phosphate deposits, related to HA, in samples before and after immersion in SBF for 14 days. The insert SEM image in Figure 8 shows the MBG surface with HA apatite related compounds after 14 days of immersion. Both MBG and MBG EOGe pellets showed the presence of P-O asymmetric bending in HA bands at 557 and 600 cm^−1^, CO_3_^−2^ bending at 874 cm^−1^ and a band with a mix component of Si-O-Si and Si-OH symmetric stretching between 1060 and 1000 cm^−1^ [46,47]. These bands clearly denote the presence of apatite-like deposits in both particles, whether loaded with EO and Ge or not, indicating that the antibacterial agents do not interfere with hydroxyapatite formation and deposition on the surface of the MBG particles. 

**Figure 9:**
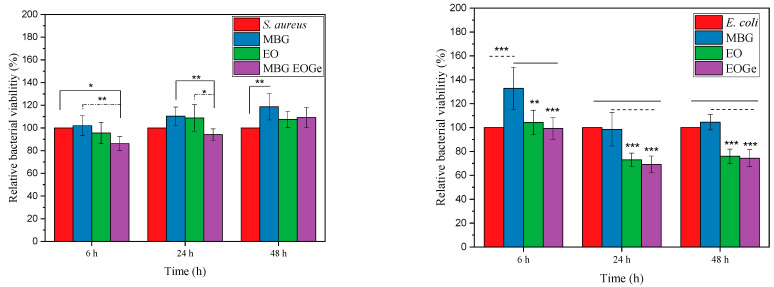
Relative bacterial viability for the MBG, MBG EO and MBG EOGe particles after 6, 24 and 48 h incubation with with *S. aureus* (Gram-positive) and *E. coli* (Gram-negative) bacteria. The asterisks indicate significant differences. * *p* < 0.05, ** *p* < 0.01 and *** *p* < 0.001.

Antibacterial activity of MBG particles could be reached in two ways: changing the composition of the bioactive glass i.e., containing Mn, Cu, Ce or Ag ions that have proved antibacterial or antifungal properties [6,22,48,49], or loading the particle with therapeutic agents such as antibiotics or essential oils. Several essential oils derived from aromatic plants, such as cinnamon, could be used as loading agents for MBG particles [50]. Other EOs with antibacterial activity are the ones containing eucalyptus related compounds. The main component of *Melaleuca armillaris* EO is 1,8-cineol [50]. This oil has proven effects against bacteria, and could also be encapsulated in different nanoparticles [51,52]. Figure 9 shows the turbidity test for *S. aureus* and *E. coli* for the MBG, MBG EO and MBG EOGe loaded particles after different incubation times. It can be clearly seen that the antibacterial effect after 24 and 48 h of the MBG EO and MBG EOGe particles is marked for *E. coli*, but not clearly noticed for *S. aureus*. One possible explanation is due to the differences in the structure and thickness of the cell membrane of both bacterial strains. Gram-positive bacteria such as *S. aureus* have a thick peptidoglycan layer and no external lipid membrane whilst Gram-negative bacteria (*E. coli*) exhibit a thin peptidoglycan layer and an external lipid membrane [53]. Situated in the outer membrane are diffusion channels (porins) through which small hydrophilic molecules, such as gentamicin, can enter the cell [54,55]. Furthermore, the results indicated that adding EOs to the MBG particles inhibited approximately 20% bacterial viability of *E. coli* compared to unloaded MBG particles. Even though the findings did not exhibit high antibacterial activity, the results suggested that adding EO into the MBG particles could enhance the antibacterial effect on *E. coli* bacteria. However, even though EO and gentamicin loaded MBG particles were effective by 48 h, there was no significant difference between the EO and EOGe samples. In a similar study, Zhong et al. [56] developed tea tree oil-loaded mesoporous silica particles. Their results showed that the incorporation of tea tree oil enhanced the antibacterial properties of mesoporous silica particles. 

Another explanation is based on the fact that dissolution of MBG particles could affect the antibacterial activity as a result of pH changes [57]. However, our results indicated that in the first 3 h MBG bacterial viability was higher than that of the control group. This could be explained by the lower dissolution ratio of MBG particles in the bacteria medium in the first 3 h of incubation. 

**Figure 10:**
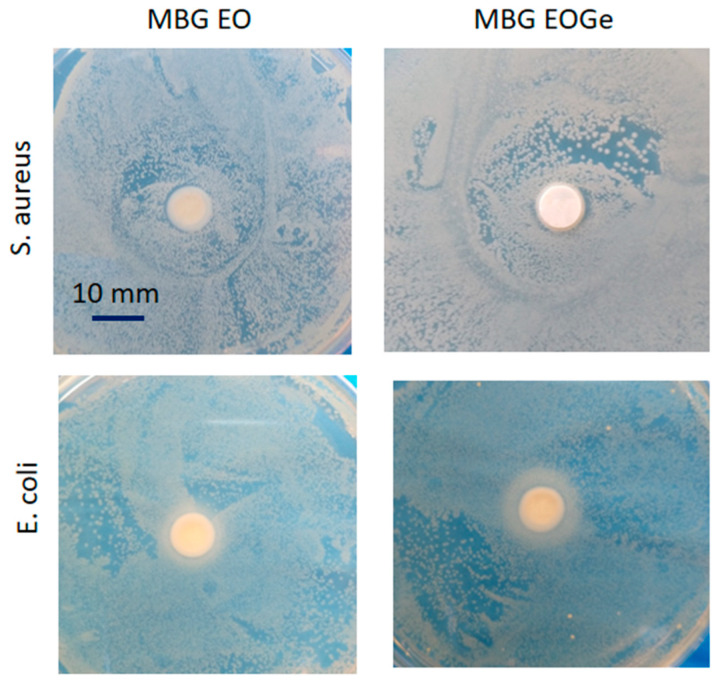
Agar diffusion test of the MBG EO and MBG EOGe pellets in contact with *S. aureus* and *E. coli* bacteria after 48 h growth.

Gentamicin is bactericidal and is a broad-spectrum antibiotic (except against *streptococci* and anaerobic bacteria). Its mechanism of action involves binding to the 30S ribosomal sub-unit, which causes the misreading of the genetic code and interrupts normal bacterial protein synthesis. This effect results in changes in the cell membrane permeability, which results in additional antibiotic uptake, further cell disruption, and finally, cell death [58]. The mechanisms of action of essential oils affect the degradation of the cell wall and cytoplasmic membrane, cytoplasm coagulation and diffusion through the double lipid layer, which affects membrane permeability and function [15,59]. Also, the dissolution and normal degradation of silica based bioactive glasses, especially those with high surface area like MBG particles, can lead to a change in the basicity of the surrounding media, creating a less viable environment for bacteria evolution and reproduction. This dissolution could be clearly seen in Figure 10, with the agar diffusion tests. In this case, the effect of the EO presence can alter the cytoplasmatic wall of gram-negative bacteria, like *E. coli*, but it is not especially effective against gram-positive ones. A synergic effect of adding gentamicin to the formulation was expected, but the effect was not sustained in time (after 48 h). Additionally, contrary to our results, Shahriarinour et al. [37] reported that thymol-loaded mesoporous silica nanoparticles increased the inhibition zone against *S. aureus* and *E. coli* bacteria. The results suggested that each EOs has a different effect on gram-positive and gram-negative bacteria, which is likely due to the variety in the main compounds.

## 4. Conclusions

Mesoporous bioactive glass particles were synthetized and characterized. The SiO_2_-CaO-P_2_O_5_ composition with 60% (mol) of silica allowed to generate 300 nm-diameter highly porous structured particles. These particles were loaded with essential oil from *Melaleuca armillaris* species, and also co-loaded with gentamicin, employing a high efficiency simple procedure. The release of the EO was fast in the first 48 h (reaching 80% of EO release) and then remained constant and low. The presence of the antibacterial agents in the particles did not affect the in vitro bioactive behaviour of the MBG particles, presenting the formation of hydroxyapatite related CaP compounds after 7 days of immersion in a fluid that simulates the inorganic composition of human plasma. The antibacterial effect of the essential oil and gentamicin was more marked for *E. coli* bacteria, but also denoted for *S. aureus* after 24 h of incubation. The presented results indicate that MBG particles with the tested composition and loaded with essential oil of *Melaleuca armillaris*, are highly bioactive and exhibit an antibacterial behaviour against gram-negative bacteria. They are therefore interesting building blocks for applications as coatings and tissue engineering scaffolds.

## Figures and Tables

**Table 1:** Value for media and SD for the major and minor axis measured for the synthetized particles, in μm.

	MBG	MBG EO	MBG EOGe
**Major axis**	0.29 ± 0.07	0.32 ± 0.08	0.30 ± 0.08
**Minor axis**	0.21 ± 0.05	0.22 ± 0.05	0.21 ± 0.05

**Table 2:** Obtained parameters and adjust (R^2^) for the release mechanism (pseudo second order).

Type of Particle System	Q_e_	k_2_	R^2^
MBG EO	36.09	7.04 × 10^−3^	0.99
MBG EOGe	24.16	5.92 × 10^−3^	0.99
MBG EOGe chit	11.05	5.15 × 10^−3^	0.98
